# Interpretable machine learning models classify minerals via spectroscopy

**DOI:** 10.1038/s41598-025-92686-2

**Published:** 2025-05-06

**Authors:** R. Smith, Tyler L. Spano, Marshall McDonnell, Lance Drane, Ian Gibbs, Andrew Miskowiec, J. L. Niedziela, Ashley E. Shields

**Affiliations:** Oak Ridge National Laboratory, One Bethel Valley Road, Oak Ridge, TN United States

**Keywords:** Machine learning, Raman spectroscopy, Uranium minerals, Material identification, Geochemistry, Mineralogy, Raman spectroscopy, Scientific data, Solid-state chemistry

## Abstract

Developing methods to identify mineral species confidently and rapidly from Raman spectral analysis is critical to numerous fields. Traditionally, analysis relies on pattern matching the Raman spectrum of an unknown dataset with a supporting library of well-characterized spectral data, which may prove difficult for environmental samples that are poorly crystalline or phase mixtures. Here, we developed interpretable machine learning models that can classify uranium minerals by secondary oxyanion chemistry and other physicochemical properties based solely on Raman spectra. This new ML method produces a mineral profile of physical and chemical properties for an unknown sample and can rapidly classify or identify unknown minerals from Raman data, without the need for an exact pattern match in a spectral library. Training models are validated by 1. Strong correlation of high confidence model regions with published spectroscopic assignments and 2. Correct classification of a mineral not present in training data. Training data are from the Compendium of Uranium Raman and Infrared Experimental Spectra and available crystallographic information files within the open-source Smart Spectral Matching scientific framework. Physically meaningful classifier models can rapidly identify key structural and chemical information about unknown uranium minerals and the overall methodology is broadly applicable for mineral phases.

## Introduction

Acid and metalliferous drainage (AMD) related to historic metal or coal mining represents a significant and complex environmental problem^1^ that can be better understood through the lens of alteration mineral speciation, although assemblages in these systems may be highly complex. Likewise, identifying the recoverability and appropriate processing pathways for ore materials may be achieved through detailed examination of mineralogical constituents of deposits, among many other structural and geochemical variables. Similarly, legacy contamination related to uranium exploration, mining, and milling has had a lasting effect on the environment^2^, with U(VI) alteration species governing the solubility and mobility of environmental U. Speciation of secondary U(VI) minerals and the resulting migration of U in the environment, and analogously, the chemical form of AMD effluent materials, and ore constituents is strongly coupled to the individual mineral species that contribute to complex assemblages. Thus, identification of mineral species^3^, remains a critical concern.

One method employed for identification of mineral species is optical vibrational spectroscopy. Fieldable techniques, such as Raman spectroscopy, can provide valuable insights into material identification, local chemical coordination environments, and bonding behavior in the solid phase with limited to no sample preparation required. However, identification of mineral species using these techniques requires large, high-fidelity libraries of reference data to identify materials via pattern matching. For minerals where such libraries are unavailable, or where materials suffer from loss of crystallinity or phase mixing, Raman and IR spectroscopy can still be employed for phase identification in these cases to provide insight into short-range order within these materials^4^, but new approaches to Raman analysis that go beyond pattern matching are required to unambiguously determine mineral species from their spectra.

Advances in artificial intelligence and machine learning have enabled development of new tools for mineral identification. ML methods have been employed to predict mineral speciation from well logs, petrographic thin sections, and whole rock chemistry^5–7^ with applications in deposit modelling, geometallurgical processing, and predictive/ descriptive mineralogy. Likewise, spectroscopic applications of ML have recently been explored^8^ with promising implications for forensic investigations of illicit drugs. Despite these advances, limitations exist. First, many ML models lack physical interpretability, i.e., it can be difficult to attribute predicted features to real physiochemical properties. This interpretability issue can be highly non-trivial and is still an emerging field, rendering the discovery of potentially novel spectral features based on these models difficult^9^. Second, there is often a schism where expertise is divided between ML specialists, and domain scientists. Existing ML methods do not leverage physical insights available from Raman spectra to improve model training. Finally, a lack of available datasets severely limits development of ML models for specific use cases.

**Fig. 1:**
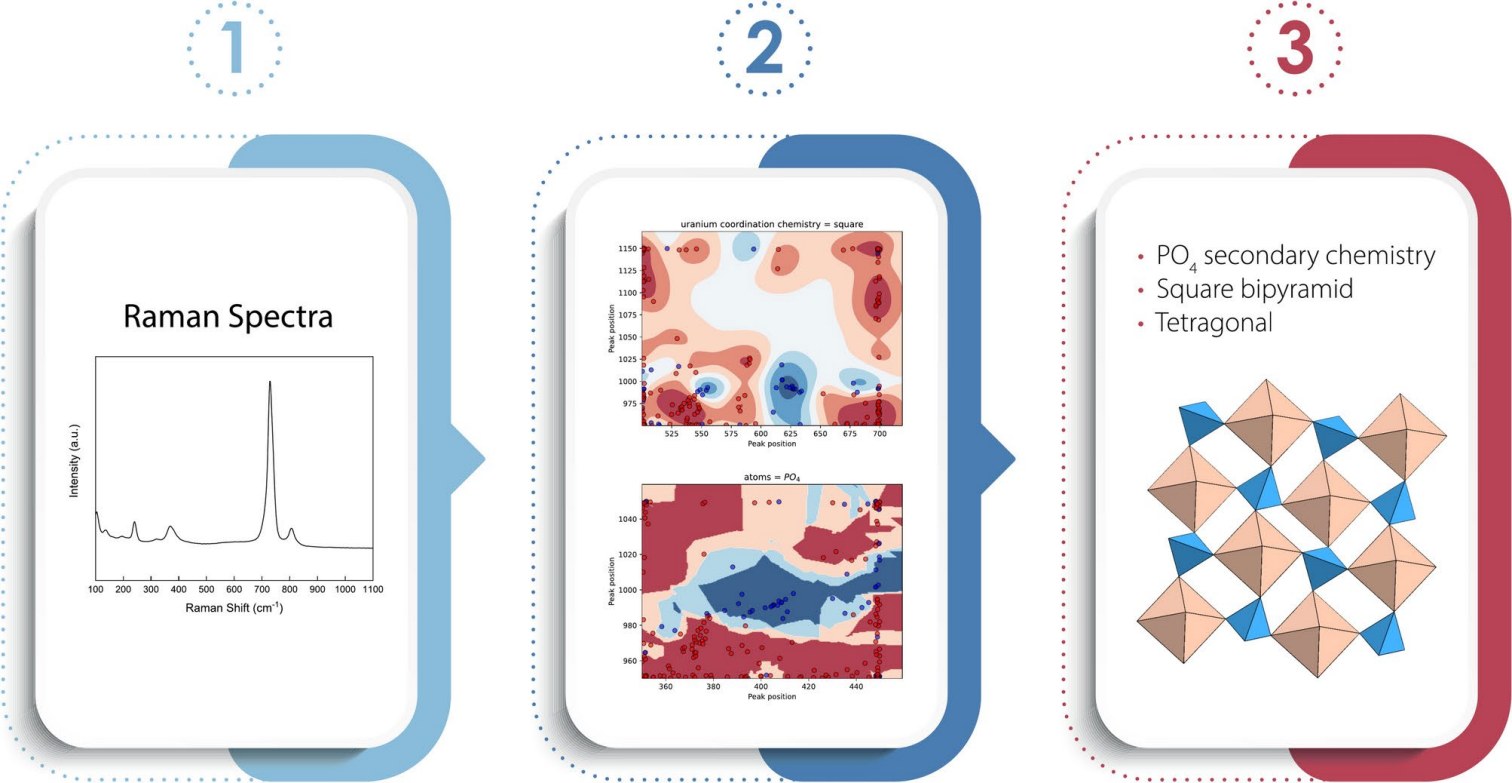
Illustration of the Smart Spectral Matching workflow: Step 1 is ingestion of raw Raman spectral data from an unknown sample, Step 2 runs this spectral data against trained classifier models to return a present/absent determination with associated confidence, generating a mineral profile in Step 3 with chemical and structural information.

The focus of this work is development of models for identification of U mineral alteration products produced via oxidation hydration weathering of the primary ore mineral uraninite (UO_2+x_), although the techniques presented herein are readily extensible to other mineral systems. For model training and validation, we use the data contained in the recently reported Compendium of Uranium Raman and Infrared Experimental Spectra (CURIES)^10^, the largest existing dataset of Raman spectra and associated crystallographic information files for uranium minerals. Specifically, we use the spectral features and structural information contained in CURIES^10^ to define classifiers, and use these classifiers to develop and train ML models able to assign samples correctly to a given class. In our method, a *classifier* is a model predicting whether a sample contains the given *class* with an assigned confidence. Confidence is the normalized internal score that the particular ML algorithm (e.g., nearest neighbor, Gaussian process, etc.) calculates to decide a sample’s class assignment. A *class* may be a secondary oxyanion species or other chemical or structural feature present in the mineral. For example, the vanadate oxyanion is a *class*, and the model that predicts whether a sample contains the vanadate oxyanion is a *classifier*. Thus, rather than spectral pattern matching for an unknown, our approach aims to identify correspondences between classifier decisions (e.g., is a mineral a uranyl sulfate?) and regions of spectral features that can be understood and inform of the underlying physical, crystal, and chemical origins of potentially novel spectral features. The classifiers are stored for future applications within the Smart Spectral Matching (SSM)^11^ scientific software framework, a workflow illustrated in Fig. 1.

Care has been taken to address and overcome known issues with ML approaches to material classifications. We designed our approach to be distinct from previous ML attempts^12 ^at material identification, which focused on full spectrum matching^5–8,13^. These efforts in full spectrum matching produced high-accuracy classifiers for recognizing materials in the reference library but lack transferability to unknowns. While CURIES is the most complete data source available for uranium minerals and related phases, it is limited by the availability of spectral data, which only exists for 83 of the ~ 275 reported U(VI) minerals, making it difficult to characterize U mineral samples via full pattern matching. Thus, the full spectrum approach cannot inform characterization of potentially exotic mineral species that are absent from the database or degradation products that are similar to, but distinct from reference data. A further issue with full spectrum matching methods is the difficulty in obtaining potentially new information about spectral signatures, as a spectral similarity measure does not directly inform underlying physical structure absent additional interpretation from crystallographic or other physiochemical information. This interpretability issue can be highly non-trivial and is still an emerging field, rendering the discovery of potentially novel spectral features based on these models difficult^9^.

With the approach presented herein, we exploit CURIES to train high confidence, interpretable models with applicability outside of the training data. Even with an incomplete reference library and operating within a small data challenge, this new ML approach can produce a mineral profile of potential physical and chemical properties for an unknown sample. The approach is applicable for mineral phases beyond U(VI) species, with potential to rapidly identify an unknown mineral from its optical vibrational spectra. Indeed, we found unique indicators of mineral groups that enabled distinguishing between species based on secondary anion chemistry, however, significant input from domain scientists was required for that work^10^.

## Results and discussion

### ML accurately identifies spectroscopic features related to mineral chemistry

Instead of identifying a material with a single ML model, we trained multiple models to recognize a particular property of minerals in either a one-vs-all or binary approach, which provides performance comparable to more complex multiclass classification strategies, as discussed in the Methods^14^. The core of the approach sections Raman spectral data in the energy domain, looking for peak intensity characteristics above background intensity. The method then determines and names classifiers and conducts pairwise comparisons against different classifier schemes, retaining the top performing classifiers. The F1 score is the metric used to evaluate classifiers during this process. Each classifier has an F1 score^17^ calculated as a measure of performance (Eq. 2) based on the number (*N*) of true and false positives and negatives.

In the one-vs-all approach, one ML classifier is trained per class and a new sample is assigned the label whose class’s binary classifier returns the highest confidence. Classes investigated include secondary oxyanion chemistry and charge balancing species, uranium coordination environment, structure type, and crystal system. Uranium coordination and secondary species are binary classification scenarios, where there are two classes (a class for having the coordination chemistry and a class for lacking it) for each property, while crystal system and structure type are multi-class problems, which are addressed with the one-vs-all strategy.

A persistent challenge for ML on analytical datasets is understanding the physical origin of features that are algorithmically identified. Here, we leverage previous work^10,15^ to connect signatory spectral regions identified in the ML classifiers with spectroscopic feature assignments. The ML approach here uses the most salient feature combinations for each class, avoiding potentially confounding spectral differences between samples. Twenty-one classification models have been developed for uranium minerals and are used to build the unknown mineral profile. Eight secondary oxyanion and one charge balancing species binary classifiers have high accuracy (Table 1). Consistently, our approach positively identifies vibrational modes in excellent agreement with widely used spectroscopic assignments in a matter of seconds. It is worth emphasizing that no peak fitting or background subtraction procedures are executed in these analyses, removing uncertainties associated with peak initialization. Further, the retention of background may be a useful property of the material being analyzed and could contribute to successful identification.

**Table 1:** Accuracy for binary classifiers measured via K-cross validation.

Class	Mean accuracy ((True positives + true negatives) / # of sample across the 10 k-cross validation training sessions)	Accuracy standard deviation	Samples belonging to class
**Secondary oxyanion and charge balancing species**			
AsO_4_	0.92	0.02	22
CO_3_	0.71	0.31	27
Cu	0.75	0.06	44
H_2_O	0.94	0.06	198
PO_4_	0.86	0.06	51
SeO_4_	0.93	0.03	12
SiO	0.88	0.06	33
SO_4_	0.95	0.04	14
V_2_O_8_	0.97	0.03	28
**Uranium coordination**			
Hexagonal bipyramidal	0.92	0.05	24
Pentagonal bipyramidal	0.73	0.10	76
Square pyramidal	0.78	0.05	46

Accuracy here is the ratio of correct sample classifications to the number of samples in the test set. Mean and standard deviation are calculated from the 10 k-cross validation training sessions.

**Fig. 2:**
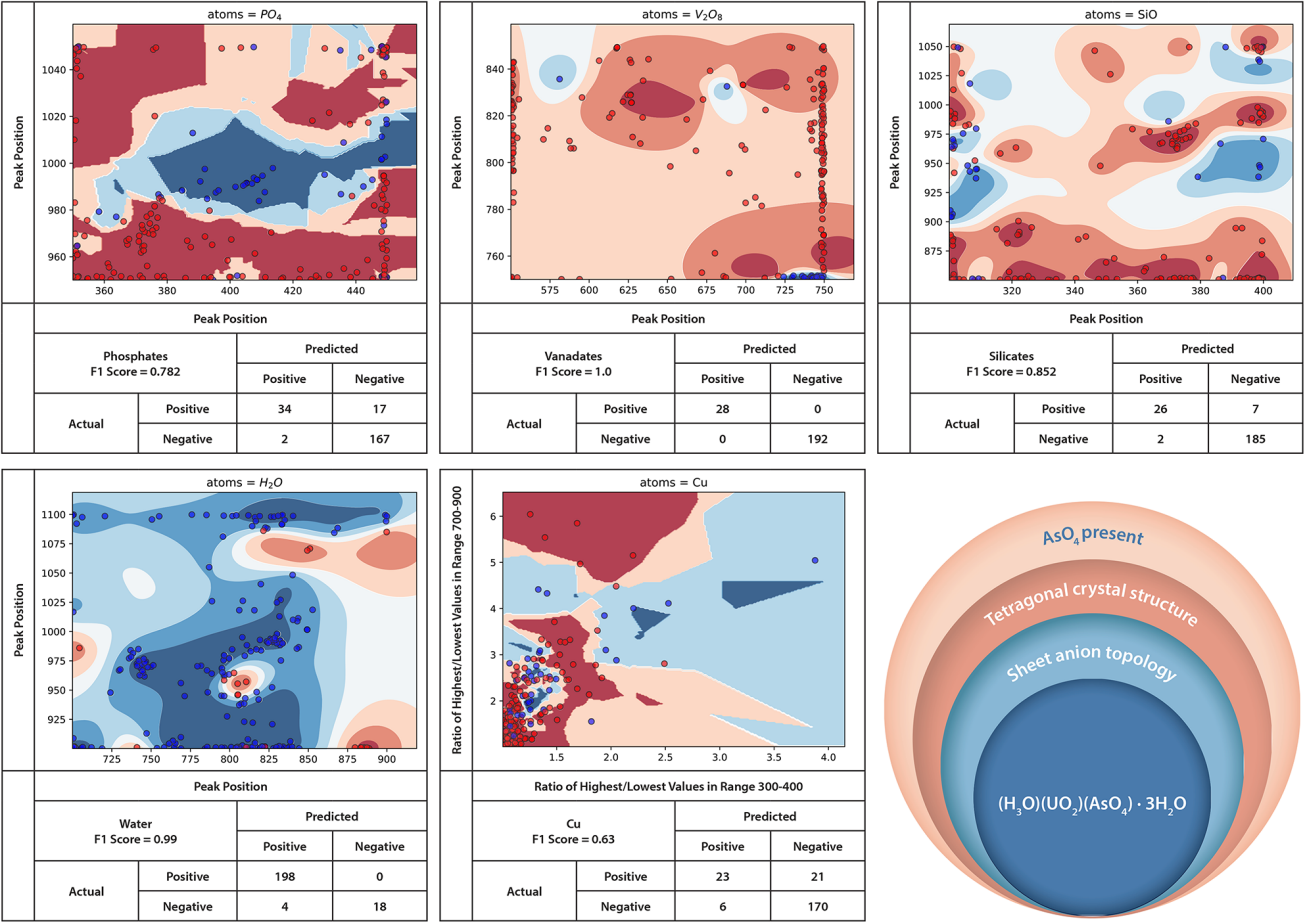
The final trained classifiers for recognizing secondary oxyanion and charge balancing species in uranium minerals from our methodology are presented for *top row, left to right: *phosphates, vanadates, and silicates, and bottom row, left to right: water, copper, and a visual representation of how these models combine to provide information on a mineral arsenate. For each model, blue dots represent training points that belong to the class while red dots represent training points that do not. The background color represents the model’s confidence that a point is (blue) or is not (red) a member of that class, with darker shades representing a higher confidence. The axes reflect the correspondence of spectral peak positions identified by the classifier training scheme, as outlined in the Methods^17^. Lower right – pedagogical depiction of classification contribution to overall mineral assignment.

The ML classifiers for uranyl phosphates, vanadates, silicates, water, and copper are presented in Fig. 2. The classifier for uranyl phosphates identifies subtle peak relationships not visible in the average uranyl phosphate spectrum of Spano et al.^10^ While the region of 975–1020 cm^−1^ has distinct spectral features in the form of intense ν_1_ and ν_3_ PO_4_^3-^ modes, our model finds a new relationship between this feature and another feature located between 360–450 cm^−1^. True positives in this region suggest that when the ν_1_ and ν_3_ PO_4_^3-^ modes are present at 975 cm^−1^, there is also a peak present at ~ 360 cm^−1^ and both peaks shift to higher frequencies in concert with the other. The model finds high confidence regions that a spectrum does *not* belong to a phosphate on either side of this narrow relationship. True positive uranyl vanadate samples are centered around features at 725–750 cm^−1^, accurately capturing the most salient feature in uranyl vanadate Raman spectra, the vibrational mode corresponding to symmetric stretching of apical vanadyl O in the V_2_O_8_ unit, centered at 737 cm^−1^^16,18^. Notably, vanadates are also characterized by the *lack* of a peak from 751–840 cm^−1^ when there is already a peak present at 725–750 cm^−1^. The silicates classifier finds high confidence regions throughout a range of 900–1050 cm^−1^, with the discriminating model features relating peaks in this range with a peak between ~ 300–310 or ~ 375–400. This range identifies high confidence regions around 900–975 cm^−1^ corresponding to the known ν_1_ SiO_4_^4-^feature^10^.The difference between simply using known vibrational modes and the model is that the model finds a new relationship between the silicate stretches and the features in the lower frequency region. No true positives have a peak between 320–370 cm^−1^ indicating peaks in this region are *not* characteristic of uranyl silicates.

Anhydrous samples are sparsely represented in the training data, but the model is still able to identify regions of moderate confidence associated with a lack of water-related features in the sample spectrum, namely in the region of 875–920 cm^−1^ vs ~ 900 or ~ 1075 cm^−1^. The hydrates, true positives, are distributed throughout the spectral regions in this model, which correlate to frequencies typically associated with U–O and U-OH vibrations. In hydrous U(VI) minerals, H_2_O groups often occupy interlayer space, acting as H bond donors or acceptors contributing to overall ionic neutrality. As a result, equatorial O atoms that coordinate U centers are often in actuality, H_2_O or OH groups^18,19^. Similarly, small quantities of bond strength from water groups can influence apical uranyl O atoms, the symmetric stretching vibrational modes of which are centered in the region of 750–900 cm^−1^^19^.

Notably, the Cu classifier, representing the only single element charge-balancing cationic species^20^ in CURIES, is the only auto-trained model for secondary chemical species in which the peak *height *in a given spectral region (defined as the ratio of the highest/lowest values in the given region) are the two features giving the highest F1 value^17^ rather than at least one feature being peak position. Very few samples without Cu had ratios of 2.0 or higher between 300–400 cm^−1^, but most Cu samples (true positives) also had ratios less than 1.5 in this region. Most of this model’s accuracy in this case appears to be the results of overfitting. Additional classifiers of secondary oxyanions are presented in the Supplementary Information.

**Fig. 3:**
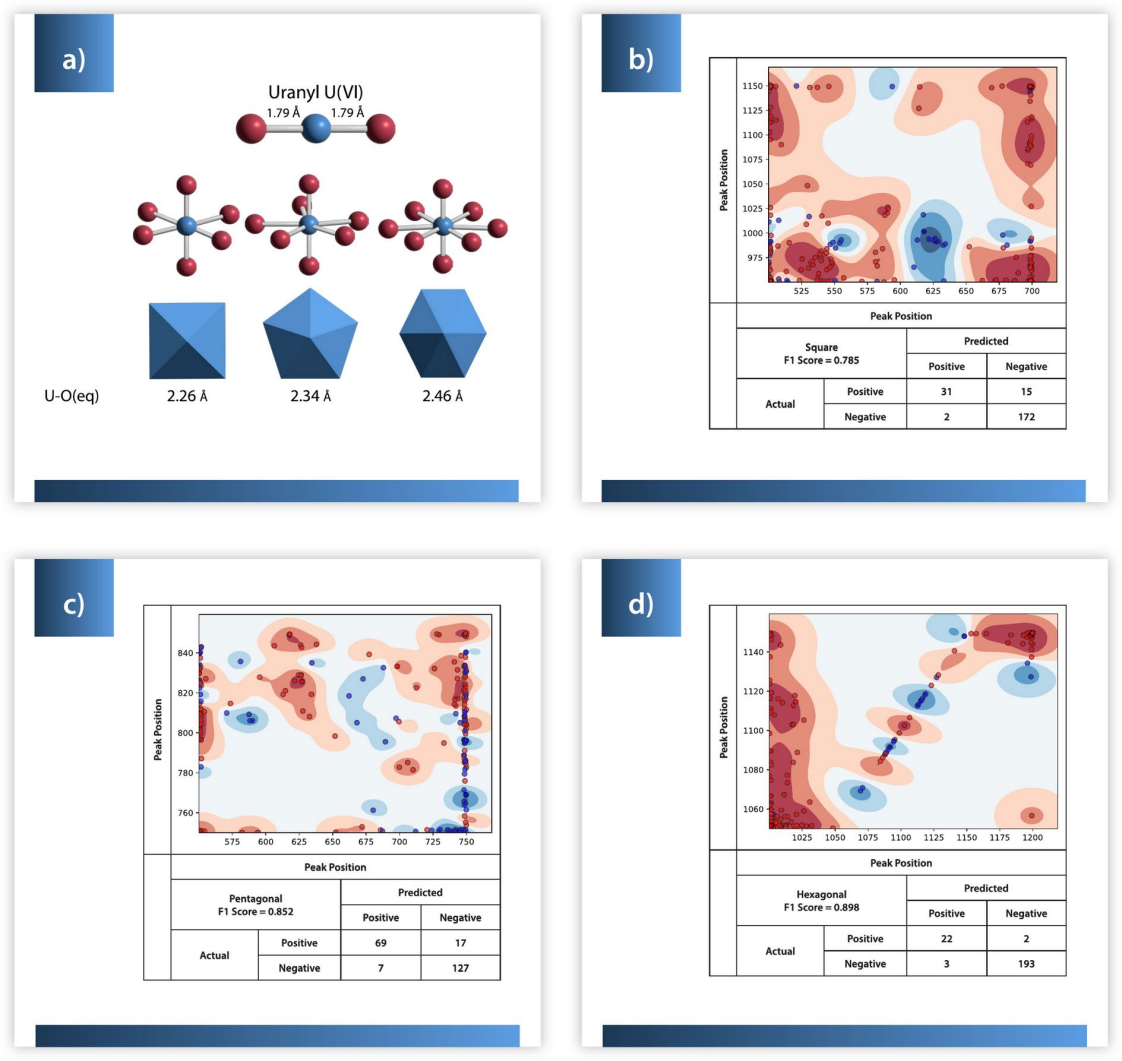
(**a**) Common coordination geometries in uranyl minerals include the linear uranyl ion (top) which forms square, pentagonal, and hexagonal bipyramids (bottom row, left to right) (**b**) Classifiers for (**b**) square bipyramidal, (**c**) pentagonal bipyramidal, and (**d**) hexagonal uranium coordination geometries^21^. Blue dots represent training points belonging to the given class while red dots represent training points that do not. The background color represents the model’s confidence that a point does (blue) or does not (red) belong to the given class, with darker shades representing a higher confidence.

The three binary classifiers for U coordination geometry (hexagonal, pentagonal bipyramidal, and square bipyramidal units^21^) have lower but acceptable performance (Table 1). F1 scores are lower than most secondary oxyanion classifiers despite large numbers of each coordination geometry represented in the dataset (Fig. 3). For square pyramidal coordination there is a cluster of true positives with peaks between ~ 610–640 vs 950–1025 cm^−1^. In general, U minerals with square pyramidal geometries do not have a peak relationship at 700 vs 950–1150 cm^−1^, with two exceptions in our data (one of which is classified correctly, and one is misclassified). The pentagonal bipyramidal model is an example of overfitting, as true positives and true negatives are quite scattered through the model regions and the model notably lacks high confidence (darkest shading) in all but a few areas where true negatives are tightly clustered. Samples with hexagonal bipyramidal U coordination do not possess Raman peaks between 1000–1050 vs 1050–1150 cm^−1^ but there are regions of low to moderate confidence that true positives have a peak at ~ 1070–1075, ~ 1090, or 1115–1120 cm^−1^. All the coordination geometry classifiers exhibit strong clustering of data with only a few points lying in regions far from large numbers of other points where overfitting occurs due to low data density.

### Rapid creation of mineral profiles made possible by suite of classification models

We build a profile of an unknown mineral to aid identification by a domain expert even if that specific mineral spectrum did not appear in the training data or reference library. As a hypothetical example, an unknown sample’s Raman spectrum could be classified by the crystal system to which it potentially belongs with the result being that the likelihood of class membership is highest for, e.g. orthorhombic. It could then be subjected to structure type and uranium coordination chemistry classifiers followed by classifiers trained to detect the presence of certain secondary oxyanion chemistries, based on the presence of spectral indicators within data regions. Thus, the classifier suite provides a profile of the hypothetical mineral as having, for example, an orthorhombic crystal system, square coordination chemistry, a sheet structure type, sulfate oxyanions, and a hydrous structure, significantly more information than currently interpretable from rapid Raman analysis of U minerals that do not meet strict pattern matching requirements. A pedagogical depiction of this is presented in Fig. 2 for the case of trögerite.

We demonstrate the approach on four minerals from CURIES that exhibit complex chemical and structural features (Table 2) that were excluded from training data. Ammoniomathesiusite, coconinoite, and schröckingerite all have multiple secondary chemistry anions and/or oxyanions present. The SSM classifiers correctly identify all of these for coconinoite but miss the presence of SO_4_ in ammoniomathesiusite and Cu in schröckingerite. While ammoniomathesiusite has vanadium present, it is in the form of VO_5_, not the V_2_O_8 _oxyanion on which we trained a classifier model, highlighting model sensitivity to the chemical environment. This sensitivity is expected, as Raman spectroscopy is a probe of chemical environment. There is no crystal structure solution available in the literature for coconinoite, but we note that the secondary oxyanions are classified correctly. Our models find that coconinoite likely possesses a triclinic unit cell but are not able to make a positive prediction as to the structure type. Trogerite is the arsenate analogue to the uranyl phosphate mineral chernikovite and the subject of renewed interest. We note there is an outstanding question as to the correct space group symmetry of trogerite^22^ but the secondary anion chemistry, U coordination, and structure type are well understood. Trögerite was correctly identified as an arsenate with moderately high confidence by our model, while the other secondary chemistry classifiers correctly returned a negative result, except for an erroneous indication of Cu present (0.67 confidence). Coordination environment analysis had two erroneous model predictions with low-moderate confidence. Broadly, from our examination of these three mineral case studies, classifiers perform best where there are the most training data, making additions to the CURIES dataset a valuable source for overall model improvement. Even with the current dataset size limitations and choosing particularly complex test minerals, the SSM classifiers can produce a largely correct and informative physicochemical profile to support identification of unknown samples purely from Raman spectroscopy.

**Table 2:**
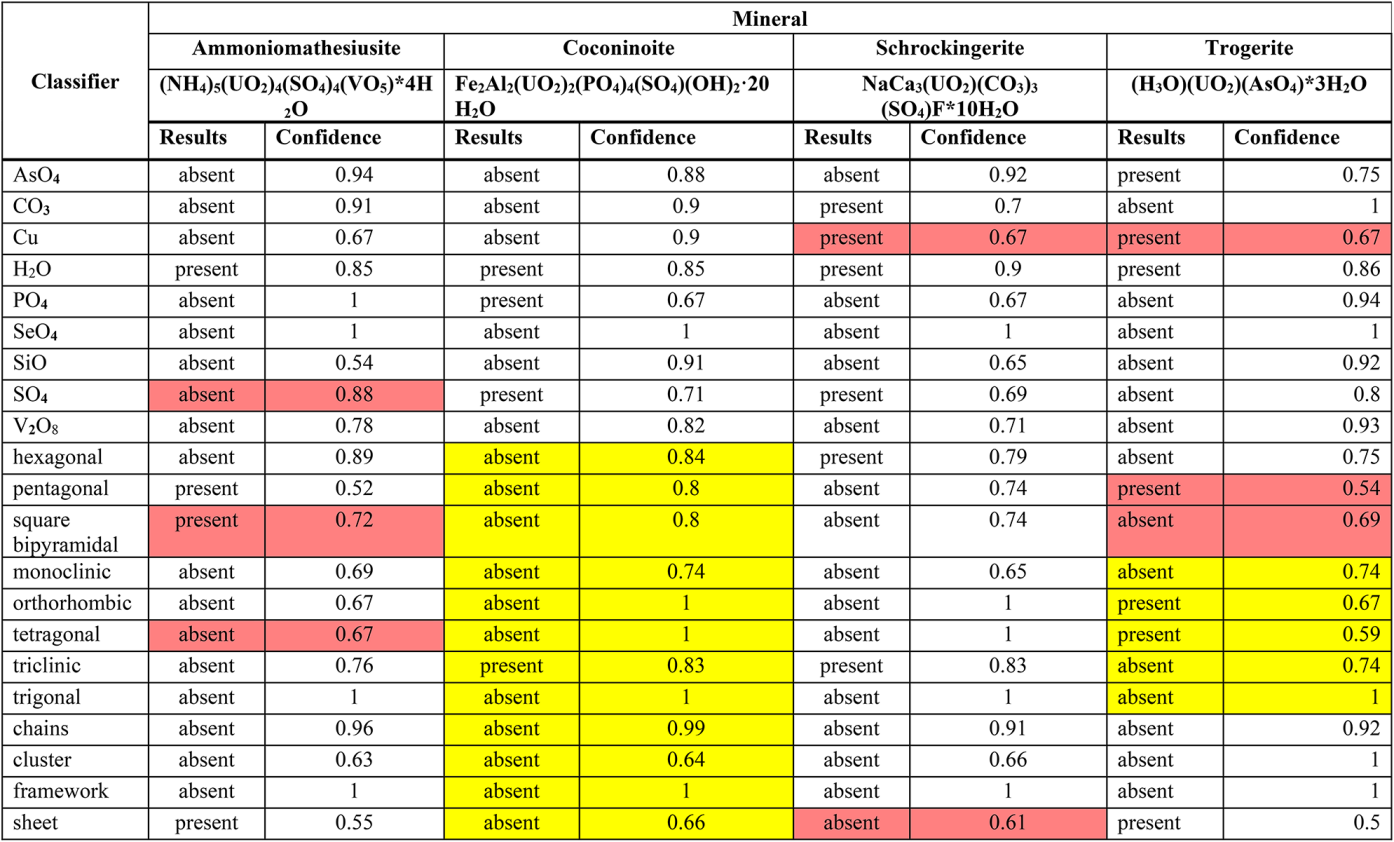
Four mineral profiles generated using SSM, with the classifier results and associated confidence in each result as calculated by that model’s specific ML algorithm (e.g., nearest neighbor, Gaussian process, etc.).

Unshaded cells indicate that the SSM model identified the *correct* structural feature while red shading indicates an *incorrect* model solution. Structural features of coconinoite are shaded y﻿ellow to indicate that there is no solved structure for this mineral; only the chemical data is available. Likewise, there is an outstanding question in the literature as to the correct space group symmetry of trögerite, so we shade this yellow as well.

## Conclusion: Rapid and transferable ML classifiers enhance potential in-field characterization

We demonstrate a ML approach to classify uranium minerals by underlying physiochemical properties reflected in optical vibrational spectroscopic features. We demonstrate the creation of a set of classifiers employing the CURIES database that can successfully train models to accurately classify unknown samples from their Raman spectra using human interpretable features. The approach does not rely on either full pattern matching, or large, complete data libraries as are required for pattern matching. Consistently, our approach correctly identifies vibrational modes in agreement with accepted spectroscopic assignments for uranium minerals, even with a training data consisting of a mix of literature reports, collected across multiple instruments at multiple institutions that would make simple pattern matching extremely difficult due to the spectral variations.

The workflow runs in a matter of seconds on a standard laptop computer. Further, the classifiers and approaches are available in the SSM platform for further development and refinement. In addition, improvement of the classifier suite is achieved by retraining when additional datasets are available. Our approach is extensible to other mineral or material groups where structural and Raman or IR data are available for training.

The capability presented here provides researchers with rapid identification for mineral unknowns based on spectroscopic attributes. The toolsets facilitate identification of phase mixtures and poorly crystalline materials. Packaging these capabilities in a lightweight software package facilitates non-destructive, in-field assignment of mineral species, with several potential applications in complex speciation associated with geological investigations.

## Method

Raman spectroscopic datasets used in this work are contained in the Compendium of Uranium Raman and Infrared Experimental Spectra (CURIES)^10^, maintained by the authors, which records all known U(VI) mineral species and contains all available spectroscopic data for U(VI) minerals. Within CURIES, features of secondary oxyanion chemistry of U(VI) minerals^23^ were detailed via compilation and subsequent multivariate analysis of Raman datasets available in the literature, including calculation and analysis of an average spectra by secondary oxyanion chemistry. In addition to spectroscopic data, CURIES also houses crystallographic information files and selected metadata pertaining to instrument parameters, additional characterization techniques, related mineral phases, relevant publications, among others. An open-source scientific data package called Smart Spectral Matching (SSM)^11^ was used to extract class labels (e.g. for U coordination, the available class labels are hexagonal, pentagonal, and square) and vibrational mode frequencies and intensities from each CURIES data set^11^.

Spectral features for training were selected based on domain knowledge of physically meaningful characteristics in Raman spectroscopy such as peak position, intensity, number of peaks, and ratio of peak intensities. Filter definitions are limited to Raman shift values between 300 cm^−1^ and 1300 cm^−1^ where all CURIES data have spectral information. Commentary on caveats on the use of peak intensities is provided at the end of this section.

Potential **features** were defined for peak intensities and positions for ranges of Raman shift starting at 300 cm^−1^. Features were defined based on peak position location and intensity above background from spectral inspection, and no peak fitting or background subtraction algorithms were used. The ranges investigate successive regions of Raman shift in amounts of 100 or 200 cm^−1^ from the starting value, producing four potential features for each pair of ranges in each energy domain:

1. peak position in the range *x* to *x* + 100 cm^−1^
2. the absolute peak height relative to average value in the range *x* to *x* + 100 cm^−1^
3. peak position in the range *x* to *x* + 200 cm^−1^
4. the absolute peak height relative to average in the range *x* to *x* + 200 cm^−1^.

The process was repeated with a new values for *x* beginning in increments of 50 cm^−1^, up to 1200 cm^−1^. To evaluate correlation between potential features and the class being investigated, the chi-squared value between the values of that feature for each point in the training data and the CURIES class label (eg. hexagonal) was calculated as:

1 $$\upchi 2 =\frac{{\left(1-\frac{\sum {N}_{values for samples in class}}{{N}_{samples}}\right)}^{2}}{\frac{\sum {N}_{values for samples in class}}{{N}_{samples}}}+\frac{{\left(1-\frac{\sum {N}_{values for samples not in class}}{{N}_{samples}}\right)}^{2}}{\frac{\sum {N}_{values for samples not in class}}{{N}_{samples}}}$$

where feature values have been normalized into the range [0, 1]^10^. The twenty-five features with the highest chi-squared values for the particular class under consideration were selected for further testing, thus eliminating from consideration spectral regions that are weakly correlated with the label.

Automated training then proceeded in two steps, 1) the best features for the singular class this model is being trained to detect are selected for training and 2) the best algorithm is chosen. To begin, initial classification of a class in question used a Gaussian process algorithm^24^. Two features from the chosen twenty-five were used pairwise for training because using three or more features caused overfitting that reduced final model performance. As a precursor to development of more accurate models, we identify feature combinations that cause members of the same class to cluster together, rather than feature combinations that require many sharp discontinuities in the classifier’s confidence to achieve a good fit. Due to the variability between samples and instruments, it is unlikely that a physically meaningful quality would identify mineral class only on the order of 1, such as vanadates only having peaks in the ranges 907–909 cm^−1^ or 912–915 cm^−1^ but never in 910–911 cm^−1^. Avoiding sharp discontinuities allows the models to capture subtle feature shifts within a given class, as is common with e.g. the uranyl peak. The Gaussian Process was chosen for this initial selection because it provides smoothness given a proper co-variance function^24^. Each classifier’s F1 score^17^ is calculated as a measure of performance (Eq. 2). The two features which together produced the highest scoring model were selected for the next step.

2 $$F1 score= \frac{2*{N}_{true positives}}{\left(2*{N}_{true positives}\right)+{N}_{false positives}+{N}_{false negatives}}$$

Finally, the two features from the preceding step were used to train nearest neighbor^25^, support vector machine^26^, multi-layer perceptron^27^, and quadratic discriminant analysis classifiers^28^. These algorithms were selected for their ability to handle training datasets with only a few hundred data points, as is the case for the CURIES database. The classifier with the highest F1 score is chosen (SI Table 3). Generally, the Gaussian process classifier produced the highest F1 score. For multi-class problems, the one-vs-all strategy is then completed by training classifiers for other classes of the chosen category by the same procedure. Analysis of the two features used in each training model compared the true positives and regions of highest model confidence against the reported spectral deconvolutions and experimental mode assignments from the literature^10^.

**Fig. 4:**
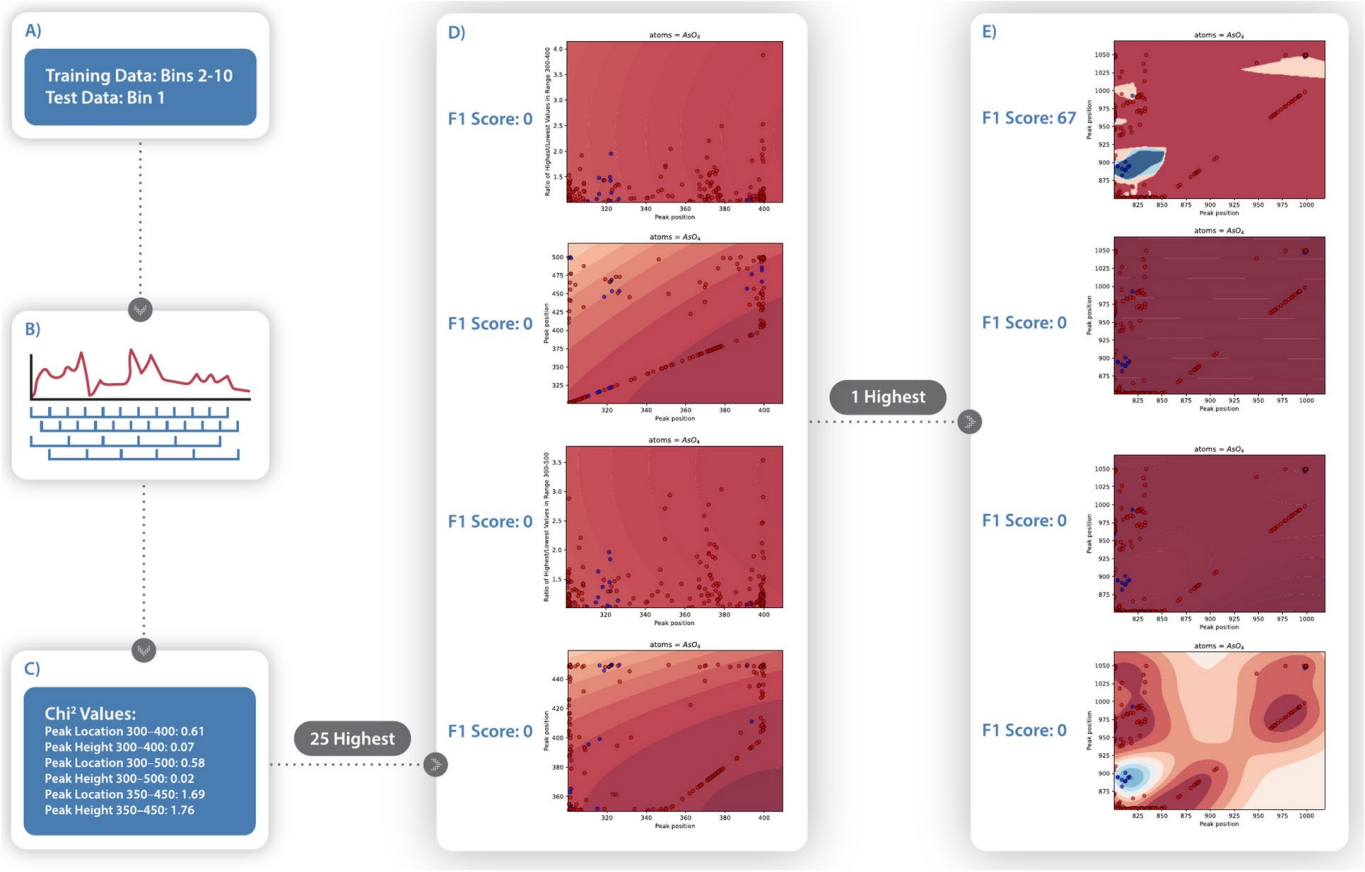
The k-cross validation procedure. A) Data is divided into 10 equally sized bins and one bin is chosen for use as test data. **B**) The wavenumber domain from 300 to 1300 cm^−1^ is divided into ranges of size 100 and size 200, starting at 300 and at 350 cm^−1^, making the first four ranges 300 to 400, 300 to 500, 350 to 450, and 350 to 550. Each range has two features defined: peak position and peak height. **C**) Each feature has a chi-squared value calculated and the features with the 25 highest values are selected for the next step. **D**) Each pair of the 25 features is used to train a Gaussian Process model and the F1 score is calculated. The two features which produce the highest F1 score are chosen. **E**) Models are trained using different techniques, using the two features chosen. The model with the highest F1 score is chosen as the final result. The process is repeated from step A, selecting a different bin as the test data, until each bin has been used as test data exactly one time.

Performance evaluation is derived from the confusion matrix. The metric used in our auto-training procedure is the F1 score^17^. We also performed k-fold cross validation^29^ a strategy often employed when datasets are too small to support a test/training split. The data is randomly partitioned into *k* equally sized groups. *K* classifiers are trained, each one using all groups but one as training data and the last as test data, recording scores for each one. Our calculations used *k* = 10, a common value above which increases in *k *no longer bring significant accuracy gains^30^. The k-fold data split was performed before the first round of Gaussian process classifier training to ensure no test data were used in feature selection. (Fig. 4).

We note that the training data in CURIES are collected across a wide variety of instruments and settings, including multiple incident wavelengths, and emphasize that the methods of classifier development do not attempt to peak fit, or background subtract either the unknown spectra or the library data. Absolute peak intensity assignment^31^ from Raman spectroscopy is known to be challenging, and subject to instrumental variability, which also contributes to failures in traditional approaches for full pattern assignment. Also, features in Raman spectra can be dispersive in the energy domain depending on the incident wavelength used in the spectrometer. Finally, relative concentration of phase mixtures can influence absolute intensities. There are no direct mitigations for any of these effects in this present work. However, the influence of these factors on the classifier performance are likely limited by the facts that (1) using database data from multiple Raman instruments and settings as training data effectively averages out these influences, (2) using peak presence or absence as supplement to peak intensities, and (3) a reasonable expectation that peak intensity variations due to changes in the instrument response function for a well-behaved Raman spectrometer will be slowly varying across the small wavenumber range defined for the classes. Addressing these challenges will be important to future work in ML developments in Raman spectroscopy in any field, particularly in the case of transferability to fielded equipment.

## Supplementary Information

Supplementary Information.

## Data Availability

Data is provided within the manuscript and will be available upon request. Please contact the corresponding author for data requests.

## References

[1] Nordstrom, D. K., Alpers, C. N., Ptacek, C. J. & Blowes, D. W. Negative pH and extremely acidic mine waters from Iron Mountain. *Calif. Environ. Sci. Technol.* **34**, 254–258 (2000).

[2] Arnold, C. Once upon a mine: The legacy of uranium on the Navajo Nation. *Environ. Health. Perspect. ***122**(2) A44–A49 (2014). 10.1289/ehp.122-A44 PMC3915248 24486697

[3] Kampf, A., Plášil, J., Kasatkin, A. & Marty, J. Belakovskiite, Na_7_(UO_2_)(SO_4_)_4_(SO_3_OH)(H_2_O)_3_, a new uranyl sulfate mineral from the blue lizard mine, San Juan County, Utah, USA. *Mineral. Mag.* **78**, 639–649 (2014).

[4] Ilieva, A., Mihailova, B., Tsintsov, Z. & Petrov, O. Structural state of microcrystalline opals: A RAMAN spectroscopic study. *Am. Miner.* **92**, 1325–1333 (2007).

[5] Maitre, J., Bouchard, K. & Bédard, L. P. Mineral grains recognition using computer vision and machine learning. *Comput. Geosci.* **130**, 84–93 (2019).

[6] Laalam, A. et al. Paper presented at the SPE Annual Technical Conference and Exhibition, Houston, Texas, USA, October 2022. 10.2118/210336-MS

[7] Kalashnikov, A., Pakhomovsky, Y. A., Bazai, A., Mikhailova, J. & Konopleva, N. Rock-chemistry-to-mineral-properties conversion: Machine learning approach. *Ore. Geol. Rev.* **136**, 104292 (2021).

[8] Madden, M. G. & Ryder, A. G. Machine learning methods for quantitative analysis of Raman spectroscopy data. In *Opto-Ireland 2002: Optics and Photonics Technologies and Applications.* 1130–1139 (SPIE).

[9] Montavon, G., Samek, W. & Müller, K.-R. Methods for interpreting and understanding deep neural networks. *Digit. Signal. Process.* **73**, 1–15 (2018).

[10] Spano, T. L. et al. CURIES: Compendium of uranium Raman and infrared experimental spectra. *Am. Miner.* **108**, 2219–2233 (2023).

[11] McDonnell, M. *et al.* Smart Spectral Matching (SSM). (Oak Ridge National Lab.(ORNL), Oak Ridge, TN (United States), 2022).

[12] Carey, C., Boucher, T., Mahadevan, S., Bartholomew, P. & Dyar, M. Machine learning tools for mineral recognition and classification from Raman spectroscopy. *J. Raman Spectrosc.* **46**, 894–903 (2015).

[13] Fu, W. & Hopkins, W. S. Applying machine learning to vibrational spectroscopy. *J. Phys. Chem. A* **122**, 167–171 (2018). 29211476 10.1021/acs.jpca.7b10303

[14] Rifkin, R. & Klautau, A. In defense of one-vs-all classification. *J. Mach. Learn. Res.* **5**, 101–141 (2004).

[15] Spano, T. L., Olds, T. A., McDonnell, M., Smith, R. & Shields, A. E. Raman spectroscopic investigation of selected natural uranyl sulfate minerals. *Am. Miner.* **109**, 274–285 (2024).

[16] Frost, R. L., Čejka, J., Weier, M. L., Martens, W. & Henry, D. A. Vibrational spectroscopy of selected natural uranyl vanadates. *Vib. Spectrosc.* **39**, 131–138 (2005).

[17] Fawcett, T. An introduction to ROC analysis. *Pattern Recognit. Lett.* **27**, 861–874. 10.1016/j.patrec.2005.10.010 (2006).

[18] Schindler, M. & Hawthorne, F. C. The stereochemistry and chemical composition of interstitial complexes in uranyl-oxysalt minerals. *Can. Miner.* **46**, 467–501 (2008).

[19] Hawthorne, F. C. The role of OH and H2O in oxide and oxysalt minerals. *Zeitschrift für Kristallogr.-Crystall. Mater.* **201**, 183–206 (1992).

[20] Hawthorne, F. C. A bond-topological approach to theoretical mineralogy: Crystal structure, chemical composition and chemical reactions. *Phys. Chem. Miner.* **39**, 841–874 (2012).

[21] Burns, P. C., Ewing, R. C. & Hawthorne, F. C. The crystal chemistry of hexavalent uranium: Polyhedron geometries, bond-valence parameters, and polymerization of polyhedra. *Can. Miner.* **35**, 1551–1570 (1997).

[22] Sweet, T. F. M. et al. Understanding the hydronium cation in the solid-state: A study in synthetic hydronium uranyl phosphate and arsenate mineral systems and their irradiation stability. *Inorg. Chem.* Under review. 10.1021/acs.inorgchem.5c00709 40630038

[23] Lussier, A. J., Lopez, R. A. & Burns, P. C. A revised and expanded structure hierarchy of natural and synthetic hexavalent uranium compounds. *Can. Miner.* **54**, 177–283 (2016).

[24] Rasmussen, C. E. & Williams, C. K. *Gaussian processes for machine learning*.**1**(Springer, 2006).

[25] Mucherino, A. et al. K-nearest neighbor classification.*Data Min. Agric.*10.1007/978-0-387-88615-2_4 (2009).

[26] Cortes, C. & Vapnik, V. Support-vector networks. *Mach. Learn.* **20**, 273–297.10.1007/BF00994018 (1995).

[27] Bradley, J. B. *Neural Networks: A Comprehensive Foundation*, IEEE Press Book (Macmillan College, New York, 1994) ISBN 0-02-352761-7.

[28] Ghojogh, B. & Crowley, M. Linear and quadratic discriminant analysis: Tutorial.*arXiv preprint* arXiv:1906.02590 (2019).

[29] Hastie, T., Tibshirani, R., Friedman, J. H. & Friedman, J. H.*The elements of statistical learning: data mining, inference, and prediction*.**2** (Springer, 2009).

[30] Marcot, B. G. & Hanea, A. M. What is an optimal value of k in k-fold cross-validation in discrete Bayesian network analysis?. *Comput. Stat.* **36**, 2009–2031 (2021).

[31] Choquette, S. J., Etz, E. S., Hurst, W. S., Blackburn, D. H. & Leigh, S. D. Relative intensity correction of Raman spectrometers: NIST SRMs 2241 through 2243 for 785 nm, 532 nm, and 488 nm/514.5 nm excitation. *Applied spectroscopy* **61**, 117–129 (2007). 17331302 10.1366/000370207779947585

