# Supplementary material for: Interpretable machine learning models classify minerals via spectroscopy
Source: Sci Rep. 2025 May 6;15:15807. doi: 10.1038/s41598-025-92686-2 (PMC12056053; doi:10.1038/s41598-025-92686-2)
Supplement: Supplementary file 1 — Supplementary Information. [file 41598_2025_92686_MOESM1_ESM.docx]

**Supporting Information for:** **Interpretable machine learning models classify minerals via spectroscopy**

R. Smith, T. L. Spano*, M. McDonnell, Lance Drane, Ian Gibbs, A. Miskowiec, J. L. Niedziela, A. E. Shields

[*spanotl@ornl.gov](mailto:*spanotl@ornl.gov)

Oak Ridge National Laboratory, One Bethel Valley Road, Oak Ridge, TN

**Additional Secondary Chemistry Classifiers**

Supporting Information Figure 1. Clockwise from top left: arsenates, carbonates, sulfates, and selenites.

**Classification by crystal system and structure type difficult due to small dataset**

The remaining nine classifiers were part of two separate one-vs-all strategies to classify crystal system and structure type, which are each text labels for each training point with an individual value (e.g., monoclinic system or chain structure) constituting. The crystal system classifier (SI Table *1*) showed good ability to find the correct class, with only trigonal crystal systems incorrectly predicted in 50% or more instances. Structure type classifier (SI Table *2*) predicted nearly every dataset as possessing a sheet structure. This is due to the highly imbalanced distribution of classes in the training data. Sheet structure types dominate U(VI) minerals. CURIES has 163 instances of sheet structure types, while the next highest structure type, frameworks, had only eighteen instances, leading to much lower confidence values assigned to the other classifiers on average [^31^](#_ENREF_31). This limited data also caused problems for the crystal system classifier, where only four examples of the trigonal class lead to a classifier that was underconfident in its predictions, causing the highest proportion of misclassifications. Note that the number of points for these two problems in SI Table *1* and SI Table *2* are different despite both using CURIES as training data due to removal of data points which did not have a crystal system/structure type defined in the database.

SI Table 1: Confusion matrix and F1 scores for the crystal system multi-class problem. The true class is the class for the sample from CURIES, while the predicted class is the class whose model returned the highest confidence for class membership for the sample.

| True class | Predicted class | | | | | F1 Score |
| --- | --- | --- | --- | --- | --- | --- |
|  | Monoclinic | Orthorhombic | Tetragonal | Triclinic | Trigonal |  |
| Monoclinic | 41 | 14 | 1 | 1 | 0 | 0.79 |
| Orthorhombic | 2 | 73 | 0 | 0 | 0 | 0.82 |
| Tetragonal | 1 | 5 | 26 | 1 | 0 | 0.83 |
| Triclinic | 3 | 9 | 4 | 33 | 0 | 0.79 |
| Trigonal | 0 | 2 | 0 | 0 | 2 | 0.67 |

SI Table 2: Confusion matrix for the structure type multi-class problem. The true class is the class for the sample from CURIES, while the predicted class is the class whose model returned the highest confidence for class membership for the sample.

| True class | Predicted class | | | | F1 Score |
| --- | --- | --- | --- | --- | --- |
|  | Chains | Cluster | Framework | Sheet |  |
| Chains | 5 | 0 | 0 | 11 | 0.48 |
| Cluster | 0 | 6 | 0 | 0 | 0.86 |
| Framework | 0 | 2 | 7 | 9 | 0.56 |
| Sheet | 0 | 0 | 0 | 163 | 0.94 |

SI Table 3: List of models and the final, highest-performing classification algorithm selected for each.

| Classifier | Algorithm |
| --- | --- |
| AsO**_4_** | Gaussian Process |
| CO**_3_** | Gaussian Process |
| Cu | Nearest Neighbors |
| H_2_O | Gaussian Process |
| PO**_4_** | Nearest Neighbors |
| SeO**_4_** | Nearest Neighbors |
| SiO | Gaussian Process |
| SO**_4_** | Gaussian Process |
| V**_2_**O_8_ | Gaussian Process |
| hexagonal | Nearest Neighbors |
| pentagonal | Nearest Neighbors |
| square bipyramidal | Nearest Neighbors |
| monoclinic | Gaussian Process |
| orthorhombic | Nearest Neighbors |
| tetragonal | Nearest Neighbors |
| triclinic | Gaussian Process |
| trigonal | Nearest Neighbors |
| chains | Gaussian Process |
| cluster | Gaussian Process |
| framework | Nearest Neighbors |
| sheet | Gaussian Process |
